# Testing for Nonselective Bilingual Lexical Access Using L1 Attrited Bilinguals

**DOI:** 10.3390/brainsci9060126

**Published:** 2019-06-01

**Authors:** He Pu, Yazmin E. Medina, Phillip J. Holcomb, Katherine J. Midgley

**Affiliations:** 1 Department of Psychology, Tufts University, Medford, MA 02155, USA; 2 Department of Psychology, San Diego State University, San Diego, CA 92182, USA; ymedinaalcantar@sdsu.edu (Y.E.M.); pholcomb@sdsu.edu (P.J.H.); kmidgley@sdsu.edu (K.J.M.)

**Keywords:** bilingual lexical access, language-selective access, language-nonselective access, L1 attrition, ERP, N400

## Abstract

Research in the past few decades generally supported a nonselective view of bilingual lexical access, where a bilingual’s two languages are both active during monolingual processing. However, recent work by Costa et al. (2017) brought this into question by reinterpreting evidence for nonselectivity in a selective manner. We manipulated the factor of first language (L1) attrition in an event-related potential (ERP) experiment to disentangle Costa and colleagues’ selective processing proposal versus the traditional nonselective processing view of bilingual lexical access. Spanish–English bilinguals demonstrated an N400 effect of L1 attrition during implicit L1 processing in a second language (L2) semantic judgment task, indicating the contribution of variable L1 lexical access during L2 processing. These results are incompatible with Costa and colleagues’ selective model, adding to the literature supporting a nonselective view of bilingual lexical access.

## 1. Introduction

For decades, researchers investigated the nature of bilingual lexical access during single-language processing: are bilinguals capable of processing only the target language or are both languages activated? The former view, known as language-selective access, proposes that bilinguals activate lexical units in the target language without non-target language translation activation. With language nonselective access, on the other hand, a bilingual’s two languages are activated in parallel even when processing a single language (e.g., “house” in English activates its French translation *maison* in French–English bilinguals). Determining the exact nature of bilingual lexical access remains an important question given the ramifications for its use in bilingualism research. For instance, nonselectivity was utilized as a mechanism for both cognitive and linguistic differences between bilinguals and monolinguals [1]. 

Despite a considerable amount of early research supporting the language-selective view, much of contemporary bilingualism research shows evidence for bilingual nonselectivity during single-language processing [2]. One exception to this trend is Costa et al.’s 2017 bilingual lexical access model, wherein recent evidence for language nonselectivity was re-interpreted to support the language selective view [3]. The present study evaluates this new model through the use of first language (L1) attrition, where this variable predicts different event-related potential (ERP) and behavioral priming results based on the validity of the model.

### 1.1. The Case for Language Selectivity

Early research on bilingual lexical access suggested a language-selective view of bilingual lexical processing, wherein a bilingual’s non-target language does not interfere with target language processing. This is exemplified in behavioral studies using repetition priming [4] and interlingual homograph and cognate lexical decision tasks (i.e., is this a real word in English?) [5] that failed to find cross-language effects. For instance, Kirsner et al. discovered that Hindi–English bilinguals showed repetition facilitation (faster reaction times) for lexical decisions when words were repeated in the same language and not when they were translation repetitions. This within-, but not cross-, language facilitation was interpreted as a bilingual’s ability to selectively operate in the target language without effects of previously seen direct translations of the target language. 

Later electrophysiological studies also provided evidence for bilingual language selectivity; bilinguals were unaffected by word frequency of non-target languages as captured by the N400 ERP component [6]. The N400 is sensitive to frequency, with high-frequency words eliciting smaller N400 amplitudes than low-frequency words. Rodriguez-Fornells and colleagues tested Spanish–Catalan bilinguals in an ERP go/no-go task requiring responses for Spanish words and withholding responses for Catalan words or pseudowords (nonwords that adhere to the orthotactics of a language) [6]. ERP waves demonstrated the expected N400 frequency effect for Spanish words but not for Catalan words. Additionally, the ERP difference waves between Spanish and Catalan words were indistinguishable from those between Spanish words and pseudowords, indicating that the non-target Catalan words were processed no differently than nonwords. If the bilinguals had activated their non-target language, as proposed by the nonselective access view, the experiment should have shown both N400 frequency effects for Catalan words and differences in ERP waves between Catalan words and pseudowords. The results are consistent with a selective access view, where bilinguals can successfully prevent processing of the non-target language. 

A mechanism for this language selectivity is an “input switch” which allows bilinguals to activate the target language and deactivate the non-target language based on linguistic input during language comprehension [7]. In language switching experiments, bilinguals were found to be slower at reading and judging the validity of sentences containing a language switch (e.g., a red-colored French word in the middle of an English sentence) than those without switches [7]. This delay in the presence of a switch was thought to reflect the process of selecting and operating in only the target language. Moreover, the authors proposed the “input switch” process to be automatic since bilinguals were slower for fixed-position switches (e.g., every fourth word in the sentence was a switch) than for random position switches, a manipulation that evaluated the effects of conscious awareness of incoming language switches on switching times. Knowledge and anticipation of future language switches appeared to have disrupted “input switch” processing as bilinguals may have tried to engage both language systems simultaneously, increasing the difficulty of the task.

### 1.2. The Case for Non-Selectivity

While the previously mentioned bilingual experiments show results consistent with the language-selective view, much of the work on bilingual lexical access suggests evidence of language nonselectivity where both target and non-target languages are activated in parallel [8,9,10]. A classic example of non-target language activation comes from the bilingual Stroop task, where bilinguals experience interference naming colors in a target language when the printed words are in the non-target language [11]. This cross-language interference indicates an inability to block out the non-target language during target language production. Other paradigms using homographs (orthographically identical words with different meanings in two languages) also suggest a nonselective access view. In Beauvillain and Grainger’s homograph-primed lexical decision task, bilinguals demonstrated greater cross- than within-language priming when the cross-language prime was higher in frequency (e.g., “four” has a higher frequency in English than in French) [12]. Moreover, bilinguals could not block non-target language processing even with conscious awareness of language switches, much like Macnamar and Kushnir’s early findings [7]; bilinguals experienced priming of English target words when primed with an interlingual homograph even when told that the primes where in French and targets in English (e.g., the French prime = *coin* priming the English target “money”). Bilinguals, therefore, appear to not only be sensitive to the lexical properties of non-target language words but are also unable to restrict processing to a target language alone. 

The most convincing evidence for language nonselectivity involves paradigms that do not explicitly display both target and non-target languages. In such cases, bilinguals are exposed to one language only, and any effects of the non-target language reliably show influences of parallel activation despite the monolingual setting. One way to manipulate implicit non-target language effects is using variable cross-language orthographic neighbors, words in the non-target language that differ from the target word by one letter. In lexical decision tasks, bilinguals are slower to respond to target words with higher cross-language neighbors and faster to respond to targets with higher within-language neighbors [13]. Such effects can be explained by the activation of both target and non-target language orthographic neighbors, with the former facilitating and the latter inhibiting lexical decisions. 

Of particular importance to the present paper is Thierry and Wu’s experiment of implicit non-target language effects in Chinese–English bilinguals [14]. In their experiment, bilinguals viewed and judged the semantic relationship between word pairs in English, their second language (L2). Half of the orthographically and semantically unrelated L2 word pairs were in fact orthographically related in their L1 translations. For instance, the L2 word pair of “train” and “ham” are not related in spelling or meaning, but their L1 translations contain an identical character *huo* (“train” translates to *huo che* and “ham” translates to *huo tui*). If bilingual lexical access is nonselective in nature, this implicit manipulation of the L1/non-target language (Chinese) should affect L2 processing. ERP results in the Chinese–English bilinguals indicated such an effect; the target word of the L1 translation related pairs elicited attenuated N400s (priming effect) compared to other unrelated L2 word pairs without related L1 translations. Critically, this difference was shown in monolingual Chinese and not monolingual English participants, suggesting that (1) the N400 priming effect can be attributable to lexical repetition priming, and (2) L1 translations are activated even when bilinguals are engaged in L2 only. 

### 1.3. Costa et al.’s Reinterpretation of Thierry and Wu (2007)

Although Thierry and Wu’s results were interpreted as the parallel activation of a bilingual’s two languages, a recent proposal aims to provide an alternative explanation [3]. Costa and colleagues [3] posited that the priming seen between the two unrelated L2 words reflects not the parallel activation of the L1 language system during L2 processing, but the adapted nature of the L2 lexicon as a function of learning. In their model, L1 lexical organization influences a bilingual’s L2 lexicon when L2 lexical acquisition occurs such that seemingly unrelated L2 words are connected. Therefore, the English (L2) lexicon in Thierry and Wu’s Chinese–English bilinguals differs from that of English monolinguals due to its development. More importantly, these superficially unrelated (no lexical or semantic relatedness) L2 word pairs can show priming effects without activation of the L1 language system. 

**Figure 1:**
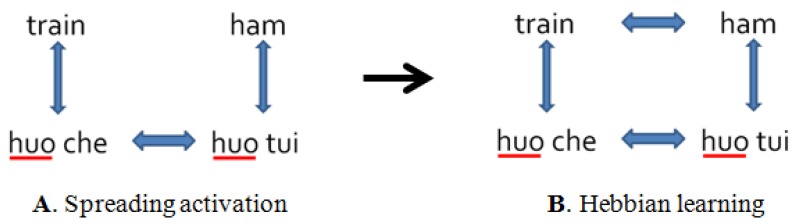
Development of the second language (L2) lexicon during L2 lexical acquisition as per Costa and colleagues [3]. (**A**) Spreading activation of four first language (L1) and L2 terms during L2 learning; (**B**) simultaneous activation of all four terms due to Hebbian learning.

However, how exactly does this L1 to L2 lexical influence occur? Costa el al. [3] utilized two concepts that take place during L2 acquisition: spreading activation and Hebbian learning. Suppose a Chinese native speaker is learning English and already knows the word “ham” (*huo tui* in L1). Upon learning the English word “train” for the first time through translation association, activation would spread to its L1 translation *huo che*. Through the same mechanism of spreading activation, *huo che* would subsequently activate huo tui (an L1 orthographic neighbor), which would then activate its L2 translation “ham” (Figure 1A). Hebbian learning (units that fire together wire together) occurs when all four items are activated so that “train” and “ham” become associated (Figure 1B). This association explains how two unrelated L2 words can prime each other without parallel L1 activation, making room for the selective view of bilingual lexical access.

Despite offering an alternative explanation for Thierry and Wu’s results, this model has a major caveat: the connections between L1 and L2 are formed during learning only. After learning is complete (e.g., when a bilingual reaches a sufficiently high level of L2 proficiency), bilinguals can selectively activate L2 lexical items during L2 processing without activation of their corresponding L1 representations [3]. Although Costa and colleagues [3] did not offer an explicit mechanism for this disconnection between L1 and L2, this proposal is not new. Other bilingual language processing models also called for the L1/L2 lexical connection separation as a function of increasing L2 proficiency (see the revised hierarchical model [15] and the developmental bilingual interactive activation model [16]). 

If L1 and L2 are in fact separated and independent after learning, then bilinguals who are fluent in L2 should no longer experience any L1 influence during L2 processing regardless of their L1 proficiency. Differences in L1 lexical access could, thus, be used to evaluate the two competing explanations for unrelated L2 priming (Costa et al.’s selective access account and the traditional nonselective access account [3]). 

### 1.4. L1 Attrition as a Method of Dissociation

An attractive method of manipulating L1 lexical access is through testing bilinguals with variable levels of L1 attrition. L1 attrition refers to the internal restructuring of the L1 lexicon that could lead to L1 vocabulary loss and lexical activation reduction, occurring when bilinguals shift from L1 to L2 use in daily life [17]. This language use shift leads to a deactivation of L1 lexical units in the absence of adequate or consistent input [18], with effects spanning across multiple levels of L1 language processing (e.g., phonology, lexical, pragmatic) [17,19,20]. Evidence for attrition-based reduced lexical access was found in lexical processing paradigms such as the verbal fluency task (VFT) and picture naming [17]. The VFT requires participants to produce as many words from a set category as possible within a time limit. L1-attrited bilinguals produce fewer L1 words than controls and perform better in their L2 than L1. The same performance degradation is seen for picture naming tasks: bilinguals show reduced accuracy and longer reaction times for L1 picture naming as a function of increasing L1 attrition, captured through length of immigration residency in an L2 speaking country and decreased L1 use [21,22].

Studies of L1 attrition effects on bilingual L1 access struggled to define a clear-cut and quantifiable measure of L1 attrition due to the multitude of factors than can lead to diminished L1 language proficiency [23]. Of these factors, L2 age of acquisition (AoA), age of emigration to an L2 speaking country, and the degree of L1 use in daily life were found to correlate significantly with L1 attrition [24,25,26,27,28,29,30,31,32]. 

Research indicates drastic L1 degradation—to the extent of undetectable behavioral and neural measures of L1 processing [26]—if attrition begins before puberty [24,25]. Demonstrative evidence of a critical period for L1 attrition includes studies on Korean adoptees who moved to France between the ages of three and 10 [26,27]. These participants claimed to have forgotten their L1 and their performance on French to Korean sentence translation and Korean sentence fragment identification did not differ from those of native French speakers without Korean exposure. In fact, the adoptees’ neural activation to Korean sentences did not differ from that to unfamiliar languages (e.g., Japanese and Polish). Further experiments on age of emigration and L2 AoA in other adoptees indicate similar findings of L1 attrition [27]. Overall, the cutoff for L1 attrition appears to range from 8–13 years of age [28], with great L1 degradation prior to this age range and minimal L1 loss post [23]. To summarize, the later the L2 AoA and age of L2 emigration are, the less the degree of L1 attrition will be.

L1 use in daily life also contributes to L1 attrition [28,29]. Because L1 attrition can be attributed to language disuse, lack of regular L1 use results in reduced access to L1 lexical items and grammar [30]. Bilinguals who use their L1s infrequently show greater L1 attrition than those with more frequent use, as shown in tasks such as L1 sentence generation [31]. Progressive L1 deterioration can also occur even if L1 is used regularly in informal settings [32]. 

### 1.5. Present Study—Research Goals and Predictions

Altogether, research suggests that L1 attrition leads to diminished L1 lexical access. In Costa et al.’s proposal [3], L1 lexical organization (e.g., *huo tui* and *huo che* association) alters L2 lexical organization (e.g., “train” and “ham” association) during learning and not after, allowing bilinguals to selectively access their L2 upon reaching high L2 proficiency. If the model is correct, then L1 attrition should not affect L2 processing in proficient bilinguals, since the lexical influences from L1 disappear after L2 vocabulary learning is completed. Specifically, highly proficient L2 bilinguals should have similar L2 lexical organization established during learning, after which the L1 has little input on L2 organization due to selective L2 processing. Therefore, the model anticipates no differences in L2 processing between bilingual L1 attriters and those with proficient L1, so long as their L2 proficiency level and learning experiences are comparable. 

The goals of the present study are twofold:

(1) Variable levels of L1 attrition in L2 fluent bilinguals will test the validity of Costa et al.’s model [3]: if L1/L2 dissociation occurs when bilinguals reach high L2 proficiency, then L2 fluent Spanish–English bilinguals should show similar levels of L2 processing regardless of their L1 attrition level.
(2) Any seen effects of L1 attrition on L2 processing would provide support for bilingual lexical nonselectivity; if L1 lexical access varies as a function of L1 attrition and such variation influences L2 language task performance, then the L1 can be assumed to be activated during L2 processing.

To address these goals, we revisited Thierry and Wu’s 2007 study by recording behavioral and ERP measures of L2 priming in Spanish–English bilinguals with varying levels of L1 attrition. The bilinguals had comparable levels of highly proficient/fluent L2 (English), but critically differed in their levels of L1 (Spanish) attrition (the factors used to derive the L1 attrition measure are detailed in Section 2). Similar to the previous study, participants performed a semantic judgment task of L2 (English) word pairs with a 2 × 2 factorial design manipulating semantic relatedness (related—e.g., “husband” and “wife”, or unrelated—e.g., “book” and “love”) and implicit L1 translation lexical relatedness (related—e.g., *hongo* and *honda*, or unrelated—e.g., *luna* and *sol*) [14]. The critical condition to the research question involved the semantically/lexically unrelated L2 word pairs that were lexically related in their L1 translations; for example, will “egg” (*huevo* in L1) prime “bone” (*hueso* in L1) in these Spanish–English bilinguals and, if so, does this priming effect differ as a function of L1 attrition? 

Predictions were made for the N400 (ERP component) priming effect based on whether bilingual lexical access is selective as per Costa et al.’s model [3] or nonselective [14]. ERPs are widely used in bilingual research due to their high temporal resolution, as well as their ability to capture effects not seen in less sensitive behavioral measures [33]. In particular, the N400 ERP component is sensitive to lexicosemantic processing, affected by lexical frequency, orthographic neighborhood size, semantic priming, and, for our study, lexical priming [33]. 

1. Costa et al.’s language-selective access view [3]: 

If Costa and colleagues’ model is correct, then the priming seen between two lexically and semantically unrelated L2 words with related L1 translations are attributable to L2 lexical organization (influenced by L1 lexical organization during learning) and not due to L1 lexical activation. In these highly proficient L2 bilinguals, no effect of L1 attrition on the L2 N400 priming effect should be seen, since L1 to L2 connections are severed after L2 acquisition.

2. Language nonselective access view [11,12,13,14]: 

L1 attrition should influence L2 processing as both languages are activated in parallel even during single-language processing. Specifically, the higher a bilingual’s level of L1 attrition is, the less L1 lexical access they have. Bilinguals with high L1 attrition should experience less L1 lexical activation during L2 processing than those with low L1 attrition, reflected in smaller N400 priming effects for the critical condition L2 word pairs (e.g., “train” and “ham”) as L1 attrition levels increase. 

## 2. Materials and Methods

### 2.1. Participants

Twenty-five Spanish–English bilinguals (17 females; ages 18–32, mean age = 22.6, SD = 3.1) recruited from San Diego State University participated in the study. All participants were native Spanish (L1) speakers and high-proficiency/fluent bilinguals of English (L2). Every bilingual learned English after Spanish and, therefore, utilized translation association L2 learning (mean age of acquisition = 8.4, SD = 3.4). All but two of the participants studied Spanish in a classroom setting, with approximately half taking classes in the United States of America (USA) and the other half studying in a Spanish-speaking country (Mexico for all but one). All participants used Spanish and English daily in speaking, writing, and reading. Their percentage of daily use of Spanish and English varied, with L1 averaging 45.6% (SD = 23.9%) and L2 averaging 65.8% (25.2%); note that these percentages did not add up to 100% due to the heavily bilingual environment of the study (a predominantly L2 environment close to the border of an L1-speaking country), where participants are frequently exposed to both their L1 and L2 in daily life. 

Participants were right-handed as per the Edinburgh Handedness Inventory [34] with normal or corrected-to-normal vision, normal psychology profile, and no history of learning or language disorders. All voluntarily enrolled in the study and were monetarily compensated for their time. All participants also provided written informed consent in accordance with the Institutional Review Board of San Diego State University.

### 2.2. Stimuli

#### 2.2.1. L1 and L2 Proficiency Measures

Participants completed the following battery of linguistic measures as an assessment of L1/L2 proficiency, history, and use:

(1) Bilingual language questionnaire—participants self-reported L1 and L2 history and use. Questions were categorized into four subsections to account for factors contributing to L1 attrition: L1 proficiency, L1 language use, L2 age of acquisition/length of residency, and L1/L2 language dominance.
(2) Shipley vocabulary test—participants completed a multiple-choice test of the best synonym for a low-frequency English word as a measure of L2 proficiency. The test contained 40 English words with four possible choices, from which participants selected the best fit meaning of the target word (e.g., “squander” = “waste”) [35].
(3) Multilingual naming test (MINT)—participants completed a Spanish and English adaption of the Boston naming test [36] as a measure of L1 and L2 proficiency [37]. Both the Spanish and English MINT contained the same 68 black-and-white pictures of items ordered in increasing difficulty (more common items with frequently used names in the beginning). The two tests differed in their language of instruction.

#### 2.2.2. ERP Stimuli

The semantic judgment task utilized 200 word pairs (prime target) of 3–11-letter English (L2) nouns with 3–10-letter non-cognate Spanish (L1) translations organized in a 2 × 2 design. Half of the pairs were lexically (morphologically and phonologically) related in their Spanish translation and the remaining half were not. Each was further halved as semantically related or semantically unrelated. The final 200 pairs consisted of four total conditions crossing semantic relatedness and L1 translation lexical relatedness (Table 1), with 50 word pairs in each of the following conditions:

(1) Semantically related/L1 translation lexically related (e.g., “fox”/*zorro* and “skunk”/*zorrillo*);
(2) Semantically related/L1 translation lexically unrelated (e.g., “thirst”/*sed* and “hunger”/*hambre*);
(3) Semantically unrelated/L1 translation lexically related (e.g., “bone”/*hueso* and “egg”/*huevo*);
(4) Semantically unrelated/L1 translation lexically unrelated (e.g., “cloud”/*nube* and “floor”/*piso*).

Twelve additional pairs (three in each condition) were made for the practice session prior to the actual task. 

The 400 English words used for the 200 word pairs were selected and translated from a corpus of over 1000 Spanish nouns derived from Mexican government-mandated Spanish textbooks (grades 4–6) [38]. Textbooks from Mexico were chosen based on the Spanish-speaking population of San Diego, the majority of whom are Mexican Spanish speakers. The grade levels were chosen due to the language experience of the study sample: native Spanish speakers who studied Spanish in a classroom setting when they were younger. Ten native Spanish speakers who were fluent in English translated the corpus from which the 200 word pairs were selected, ensuring that the L1–L2 translations were consistent across Spanish–English bilinguals and lexically similar for the translation lexically related conditions. Additionally, a pilot English semantic judgment task using fluent English speakers (*n* = 3) determined the categorization of semantic relatedness for the English word pairs (only pairs eliciting unanimous “yes”/“no” responses were considered).

The prime and target for each word pair were matched for their English length, Spanish translation length, grade level, frequency, English orthographic and phonological neighborhood [39], and concreteness. Additionally, all four conditions were matched on these metrics. The 200 L2 word pairs were then randomized and split into two blocks of 100 word pairs (25 for each condition), with presentation order counterbalanced across participants (see Table S1 under Supplementary Materials for a list of critical stimuli)

#### 2.2.3. Post-ERP L2 to L1 Translation Task

In order to verify the ability of the participants to correctly translate the L2 word pairs into their respective L1 translations (a necessity to validate the lexical relatedness condition), participants completed a translation task of all seen stimuli after completion of the ERP task. This task showed all 400 English words from the task in randomized order, and participants were asked to translate each word into Spanish. 

### 2.3. Procedure

#### 2.3.1. Screening

All participants completed the L1 and L2 proficiency measures (described in Section 2.2.1) as part of screening procedures. Two online platforms (Qualtrics survey software [40] for the questionnaire and Testable [41] for the three linguistic tests) were used for the measures, and links were sent to potential participants. On the Qualtrics-based bilingual questionnaire, participants answered free response and Likert-scale questions about their L1/L2 language history and use. For the Shipley vocabulary test, participants saw a capitalized target word on the center of the screen with four answer choices below, one of which they clicked to select the best synonym for the target word. The English and Spanish MINT displayed a black-and-white line drawing on each page with a free response textbox below. Participants typed the name for each picture in the respective language of the test. Language order of the two MINTs was counterbalanced across all potential participants to prevent repetition effects.

Eligibility was determined based on (1) ERP study requirements (18 years of age or older, right-handed, normal or corrected-to-normal vision, and normal psychological/neurological profile with no history of language or learning disorders), (2) bilingual sample requirements (native Spanish speakers who learned English after Spanish with high proficiency/fluency in English), and (3) score cutoffs for the Shipley vocabulary test and MINT to ensure high L2 proficiency and variable L1 proficiency (L1 attrition). The Shipley vocabulary test cutoff for adequate L2 proficiency was a score of 21 out of 40, derived from previous research on English monolinguals, English-dominant bilinguals, and balanced (equally fluent in English and another language) bilinguals [37,42,43]. The MINT cutoffs were based off previous research on Spanish–English bilinguals using the MINT [37,43]: 75% accuracy for English MINT and 60% accuracy for Spanish MINT. The lower accuracy cutoff for the Spanish MINT reflects the varying levels of L1 proficiency/L1 attrition needed for the study, as well as the understandably lower L1 proficiency in bilinguals residing in an L2 speaking country. 

#### 2.3.2. L1 Attrition Score

Each participant obtained an L1 attrition score based on results from the screening questionnaire and language tasks. The scores were derived from four categories of factors contributing to L1 attrition [24,25,26,27,28,29,30,31,32]:

(1) L1 proficiency—calculated from Spanish MINT accuracy and self-ratings of L1 reading, L1 speaking, and L1 comprehension. Higher ratings/accuracy correspond to a lower L1 attrition score.
(2) L1 language use—calculated from time spent in an L1 environment (percentage of life spent in an L1-speaking country) and percentage of daily L1 use. Longer L1 environment time spent and higher percentage of daily L1 use correspond to a lower L1 attrition score.
(3) L2 AoA and environment—calculated from L2 age of acquisition (the older the age of learning English is, the lower the L1 attrition score is), time spent in an L2 environment (the higher the length of residency (LOR) is, the higher the L1 attrition score is), and percentage of daily L2 use (the higher the daily L2 use is, the higher the L1 attrition score is).
(4) Language dominance—calculated by self-rating of language dominance on the bilingual language questionnaire and the difference between the English and Spanish MINT accuracies, with greater English (L2) dominance corresponding to a higher L1 attrition.

Each category accounted for 25 points (for a potential total of 100 points) to provide a quantifiable L1 attrition score that weighted each factor evenly. The individual L1 attrition scores allowed for correlation analyses of participant results. Additionally, the scores were used to divide participants into low versus high attrition groups for behavioral and ERP group analyses. 

#### 2.3.3. ERP L2 Semantic Judgment Task

ERPs were measured while participants completed a semantic judgment task for each sequentially presented L2 word pair (i.e., “Are these two words related in meaning?”). Participants pressed the left or right shoulder gamepad button if the target word was related to the previous word and the other button if not (“yes”/“no” buttons were counterbalanced across participants). After each decision, the next word pair was shown. Note that the L1 translation lexical relatedness manipulation was implicit and, therefore, participants never saw any Spanish stimuli during the ERP experiment. 

The 200 total word pairs were shown in two blocks of 100 in pseudorandomized order (no more than three pairs of the same condition in a row), with each block containing 25 pairs in each condition. A practice block of 12 word pairs (three in each condition) was presented prior to the experiment in order for participants to familiarize themselves with the task and button presses. For the purposes of ERP recording, participants were instructed to remain still and minimize blinking when each word appeared on the screen. 

**Figure 2:**
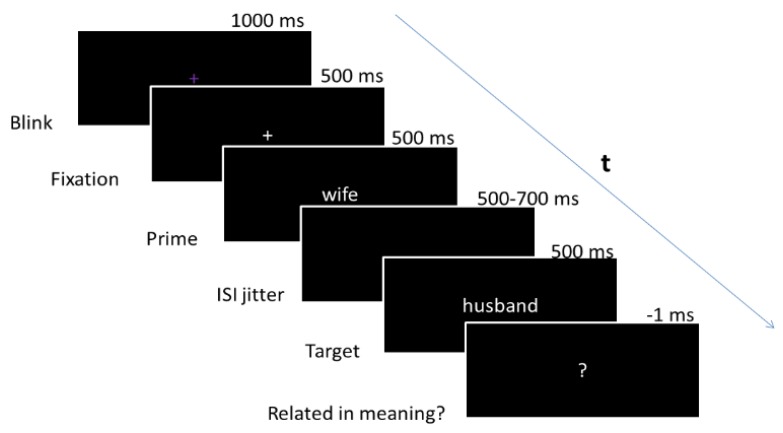
Sample trial across time (t) from the semantic judgment task while event-related potentials (ERPs) were recorded.

All words were presented in white Arial font in the center of a black background on a stimulus monitor approximately 140 cm in front of the participant. Each trial (word pair) (see Figure 2) initiated with a 1000-ms centered purple fixation cross to direct attention to the screen and allow for blinks, followed by a 500-ms white fixation cross and 200-ms interstimulus interval (ISI). The prime (first word in each pair) then appeared for 500 ms, followed by a jittered ISI of 500–700 ms (to minimize habituation or expectation [44]. The target appeared next for 500 ms, followed by a question mark. The question mark remained on the screen until the participants made a semantic judgment decision by pressing one of the two shoulder buttons, after which the next trial began. 

#### 2.3.4. Electroencephalogram (EEG)/ERP Recording Procedure

**Figure 3:**
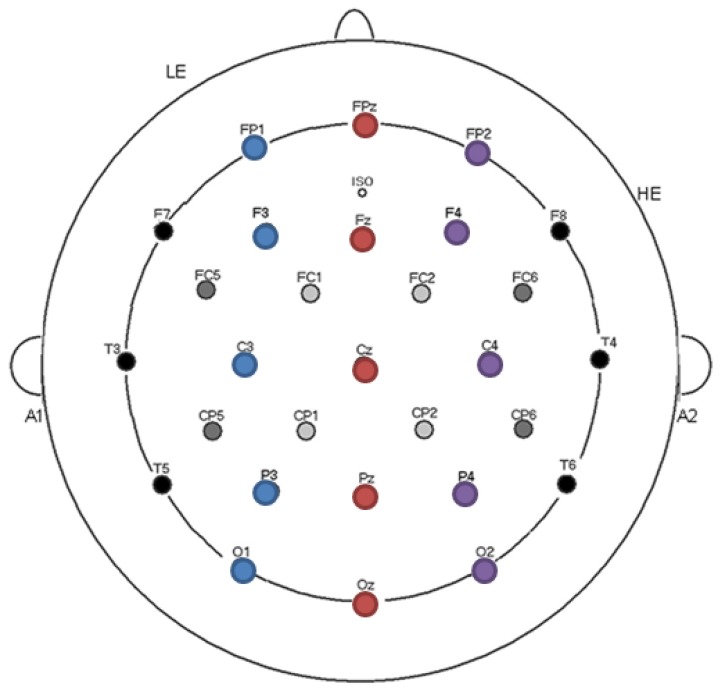
Electrode montage and electrode sites used for analyses.

ERPs were recorded from a 29-channel electrode cap (electro-cap) while participants sat in a chair in a light and sound attenuated room. Electrode positioning on the cap was based on the 10–20 electrode positioning system (Figure 3). Additionally, loose electrodes were attached behind both mastoid processes for referencing the brainwaves, as well as under the left eye (blink monitoring) and to the right of the right eye (eye saccade monitoring). Electrode impedances were kept below 2.5 kΩ except for the eye electrodes (<5 kΩ). EEG was continuously sampled at 500 Hz during the experiment and amplified at a bandpass of DC to 100 Hz using SynAmsRT amplifiers (Neuroscan). ERPs were referenced to the left mastoid electrode (A1 in Figure 3) and the ground channel positioned on the fronto-central scalp (ISO in Figure 3).

#### 2.3.5. Post-ERP L2 to L1 Translation Task

Approximately 1–2 weeks after the ERP session, participants came back for a behavioral follow-up translation task. Using Excel, they viewed each of the 400 English nouns previously seen in the ERP task and typed the Spanish translation for these words. The instructions explicitly stated that these words were all nouns and that participants should type the first Spanish translation that came to mind for each word. Translations were corrected against the original Spanish stimuli used to create the 200 English word pairs. Only correctly translated prime–target pairs were used for subsequent analyses to maintain validity in the translation lexical relatedness condition (i.e., if a participant could not translate one or both English word(s) into its designated translation(s), the prime and target would no longer be lexically related in their L1 translation, and we should not expect to see any priming effects attributable to that).

#### 2.3.6. ERP Analyses

ERPs time-locked to the onset of each target word were averaged together based on stimuli condition with a −100 ms to 0 ms baseline and a low-pass filter (15 Hz). No trials with ocular artefacts (blinks or saccades) were included in the average (rejecting 9.6% of trials across participants). The N400 component mean amplitude was calculated from averaging the EEG from 300–500 ms, a time range that corresponds with the negative deflection in the brainwave reflective of this component [45]. Analyses included 15 electrodes spanning the anterior to posterior scalp with laterality as a factor (see Figure 3): prefrontal (FP1, FPz, FP2), frontal (F3, Fz, F4), central (C3, Cz, C4), parietal (P3, Pz, P4), and occipital (O1, Oz, O2). We completed analyses of variance (ANOVAs) using a between-subject factor of attrition (low versus high) and a within-subject factor of semantic relatedness (related versus unrelated) and L1 translation lexical relatedness (related versus unrelated). 

In addition, the N400 effect for the critical comparison (effect of L1 translation lexical relatedness for semantically unrelated prime–target pairs) was calculated by subtracting the average voltage between 300 and 500 ms for targets whose translations were lexically related from those whose translations were not lexically related in the semantic unrelated conditions (see Table 1). The resulting N400 effect was used in correlation analysis against individual L1 attrition scores, as well as a group analysis between low and high L1 attrited bilinguals. All repeated measures with more than one degree of freedom in the numerator underwent the Greenhouse and Geisser correction [46]. 

#### 2.3.7. Behavioral Analyses

Behavioral statistics on accuracy and reaction time (RT) of the task were conducted between the independent variable of L1 attrition score and the four conditions. We also assessed L1 attrition against two additional dependent variables: the semantic priming effect due to the nature of the experiment (semantic judgment) and, importantly, the L1 translation lexical priming effect. The latter effect was captured in Thierry and Wu (2007) and is thought to reflect nonselective bilingual lexical access. The semantic priming effect was constructed by subtracting the average RT and accuracy for the semantically related conditions from that of the semantically unrelated conditions. The L1 translation lexical priming effect for semantically unrelated pairs (accuracy and RT differences between lexically unrelated translations of prime–target pairs which were semantically unrelated and lexically related translations of prime–target pairs which were semantically unrelated). 

Analyses included pairwise correlations (L1 attrition scores and the four conditions/semantic and L1 translation lexical priming effects) and mixed ANOVAs. ANOVAs utilized a between-subject factor of attrition (low versus high) and within-subject factor of semantic relatedness (related versus unrelated) and L1 translation lexical relatedness (related versus unrelated). Additionally, a two-way repeated measures ANOVA (semantic relatedness and L1 translation lexical relatedness) was utilized to look at the overall behavioral responses elicited by the four conditions. All significant interactions were further analyzed using *t*-tests.

RT data were trimmed to reduce effects of unrelated external influences (e.g., distractions) using a combination of two methods: an absolute cutoff and relative cutoff per participant [47]. An upper cutoff of 6000 ms was used for RT analyses based on previously used response times for L2 processing, as well as methodological reviews of psycholinguistic experiments [47,48,49]. Additionally, RTs faster or slower than two standard deviations from each participant’s mean RT were discarded [47,49]. Accuracy was then scored from correct button presses for trials with acceptable RTs. 

## 3. Results

### 3.1. L1 Attrition Score

Each participant received a composite L1 attrition score based on the factors listed in Section 2.3.2. The scores for the 25 participants ranged from 27.4 (lowest L1 attrition) to 74.2 (highest L1 attrition) with a mean of 47.4, SD = 14.6. Normality calculations (skewness and kurtosis) of attrition scores were computed using JASP (version 0.8.6) [50], and participants were grouped into low and high L1 attrition groups based on a cutoff of mean attrition ±0.25 standard deviations. Mean attrition was used over median attrition score because skewness (0.383) and kurtosis (−0.786) for the dataset were within acceptable ranges for normality [51]. Comparisons between these two subsets of the 25 participants (low L1 attrition *n* = 12; high L1 attrition *n* = 8) allowed us to capture the greatest effects of L1 attrition on L2 processing. Unpaired *t*-tests demonstrated that, while L1 attrition and factors contributing to the L1 attrition score (e.g., language dominance and L2 AoA) were significantly different (*t*(18) < 0.001) between the low and high L1 attrition groups, potential confound variables such as sex and age (*t*(18) = 0.484) were not. 

### 3.2. Behavioral Results

Participants completed the semantic judgment task with an average accuracy of 85.7%, SD = 3.5%, with correct trials taking an average of 1397.5 ms (SD = 247 ms) for RT. Average accuracy on the post-ERP translation task was 73.9% (SD = 7.4%) with a significant correlation between L1 attrition score and accuracy (*r* = −0.68, *p* < 0.001). As participants’ L1 attrition level increased, the worse their L2 to L1 translation accuracy became. This was corroborated by the significant translation accuracy difference between low (78.5%) and high (68.4%) L1 attrition groups, *t*(18) = 3.54, *p* = 0.002.

Pairwise correlations showed no significant effects between L1 attrition score and behavioral results from each condition or their respective effects (e.g., L1 translation lexical priming effect for semantically unrelated pairs). Similarly, a 2 × 2 mixed design ANOVA with within-subject factors of semantic relatedness (related, unrelated) and L1 translation lexical relatedness (related, unrelated) and the between-subject factor of L1 attrition group (low versus high) did not show effects of L1 attrition group on either RT or accuracy metrics. The following behavioral results reveal the effects seen in the overall group of Spanish–English bilinguals.

#### 3.2.1. Reaction Time Results

**Figure 4:**
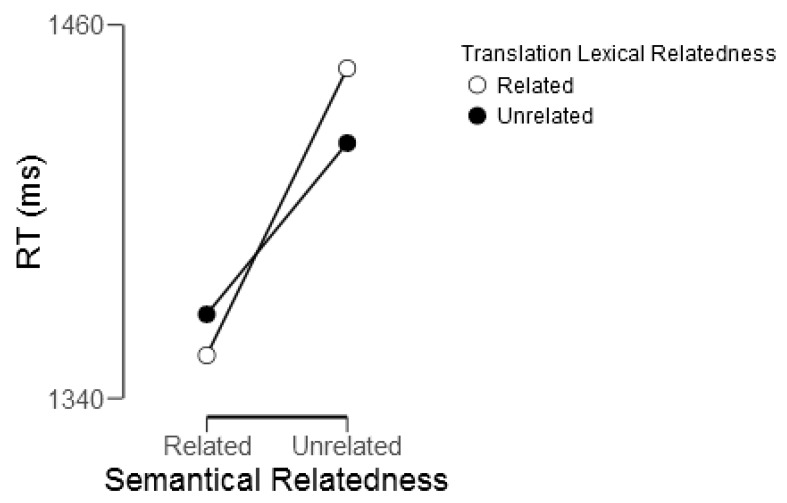
Reaction time (RT) interaction between semantic relatedness and L1 translation lexical relatedness.

Reaction time analysis revealed a main effect of semantic relatedness; participants responded to semantically related targets (mean = 1349.8 ms) faster than to semantically unrelated targets (mean = 1420.6 ms), *F*(1,22) = 13.31, *p* = 0.001. This effect was modulated by L1 translation lexical relatedness (*F*(1,22) = 9.07, *p* = 0.006); participants were faster at correctly judging semantically related targets as being related if their translations were lexically related than unrelated, and slower at correctly judging semantically unrelated targets as being unrelated if their translations were lexically related than unrelated (see Figure 4). This interaction was driven by the significant RT differences between the two semantically unrelated conditions, whereas the two semantically related conditions did not differ in reaction time. Participants responded to targets that were both semantically and translation lexically unrelated faster (1422.05 ms) than to targets that were semantically unrelated but had lexically related L1 translations (1446.02 ms), *t*(24) = 2.41, *p* = 0.02.

#### 3.2.2. Accuracy Results

**Figure 5:**
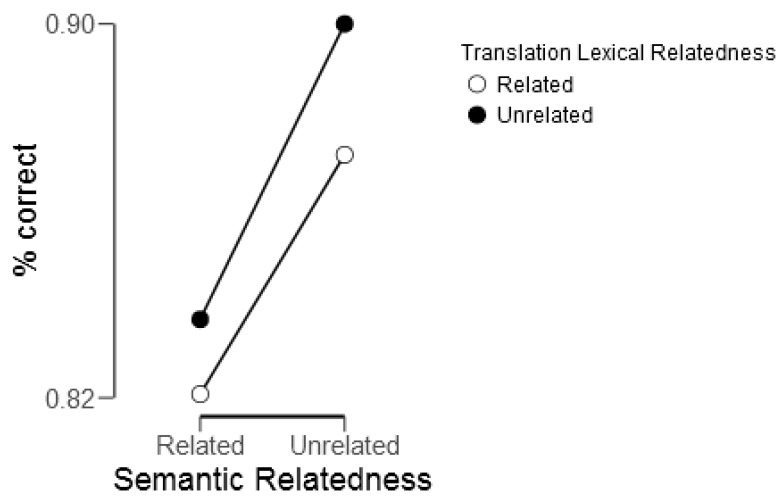
Accuracy main effects of semantic relatedness and L1 translation lexical relatedness.

Accuracy results showed the main effects of both semantic relatedness (*F*(1,22) = 8.39, *p* = 0.008) and translation lexical relatedness (*F*(1,22) = 4.298, *p* = 0.05) (see Figure 5). Participants were more accurate at judging semantically unrelated pairs as unrelated (88.4%) than semantically related pairs as related (82.7%). This is corroborated by the results of a paired *t*-test for the “no” (the two words are unrelated) and “yes” (the two words are related) responses, indicating a significant “no” response bias, *t*(24) = 3.17, *p* < 0.001. The translation lexical relatedness main effect revealed that participants were more accurate on the semantic judgment task when L1 translations were unrelated (86.8%) than related (84.6%). There was no significant interaction between the semantic relatedness and L1 translation lexical relatedness factors.

### 3.3. ERP Results

#### 3.3.1. N400 Semantic Priming Effect 

**Figure 6:**
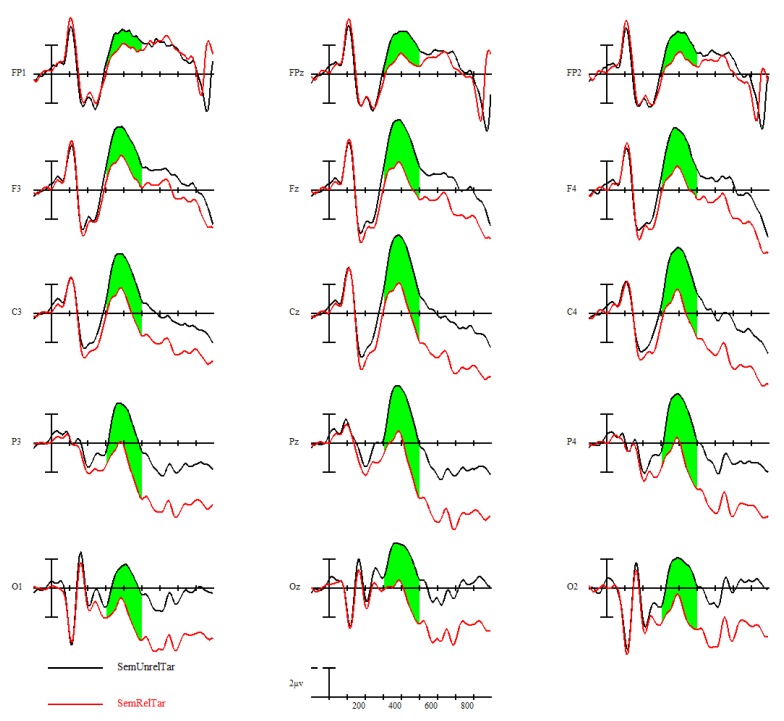
Semantic priming effect (related = black, unrelated = red) across all participants.

**Figure 7:**
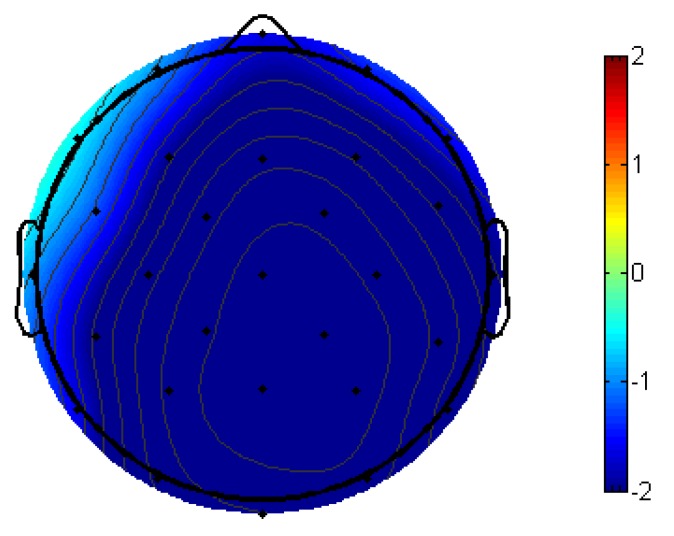
The 300–500-ms voltage map of semantic priming effect (semantically unrelated–semantically related) across all participants, with blue indicating a net negative effect.

Participants displayed a strong N400 semantic priming effect, *F*(1,24) = 43.96, *p* < 0.001; semantically unrelated targets elicited larger-amplitude N400s than semantically related targets. This is shown in Figure 6, with ERPs time-locked to target words with the black line representing semantically unrelated targets (prime was semantically unrelated to the target) and the red line representing semantically related targets (prime was semantically related to the target). A voltage map of this N400 semantic priming effect from 300–500 ms, made by subtracting the ERPs of semantically related targets from semantically unrelated targets, can be seen in Figure 7. 

**Figure 8:**
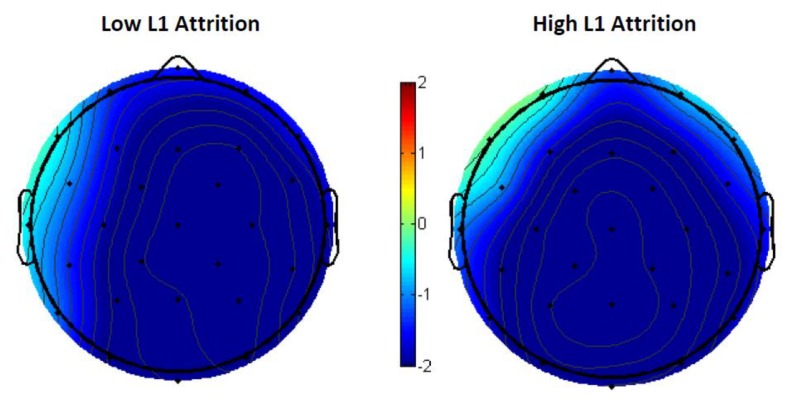
The 300–500-ms voltage maps of semantic priming effect (semantically unrelated–semantically related) between low and high L1 attrition bilinguals, with blue indicating a net negative effect.

Delving into the effects of L1 attrition, both low-attrition bilinguals, *F*(1,11) = 21.59, *p* < 0.001, and high-attrition bilinguals, *F*(1,7) = 9.47, *p* = 0.0179, demonstrated the N400 semantic priming effect, with no group by priming effect interaction (Figure 8).

#### 3.3.2. N400 L1 Translation Lexical Priming Effect 

Participants showed no main effect of an N400 L1 translation lexical priming effect in either grand mean (all participants) or attrition group comparisons.

#### 3.3.3. N400 L1 Translation Lexical Priming Effect in Semantically Unrelated Conditions

**Figure 9:**
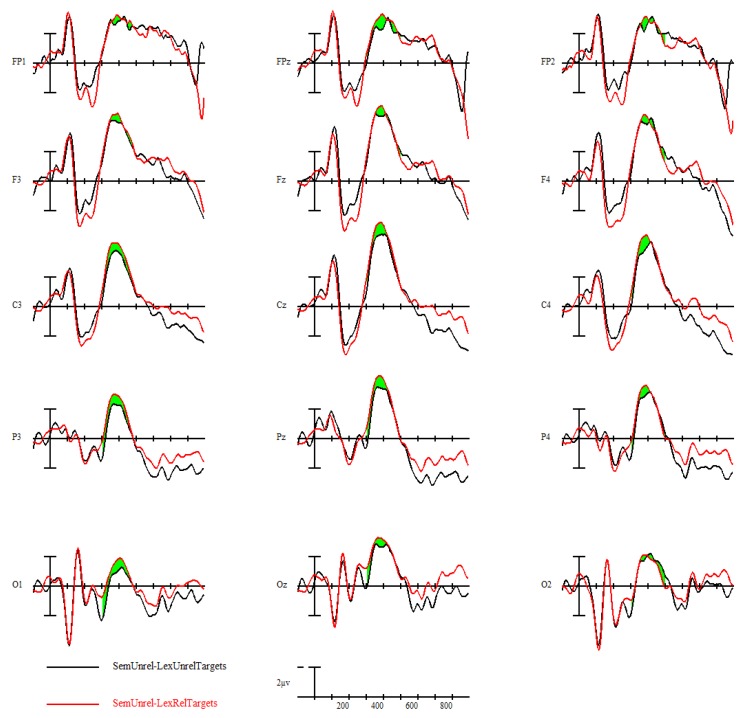
L1 translation lexical priming effect in semantically unrelated conditions.

**Figure 10:**
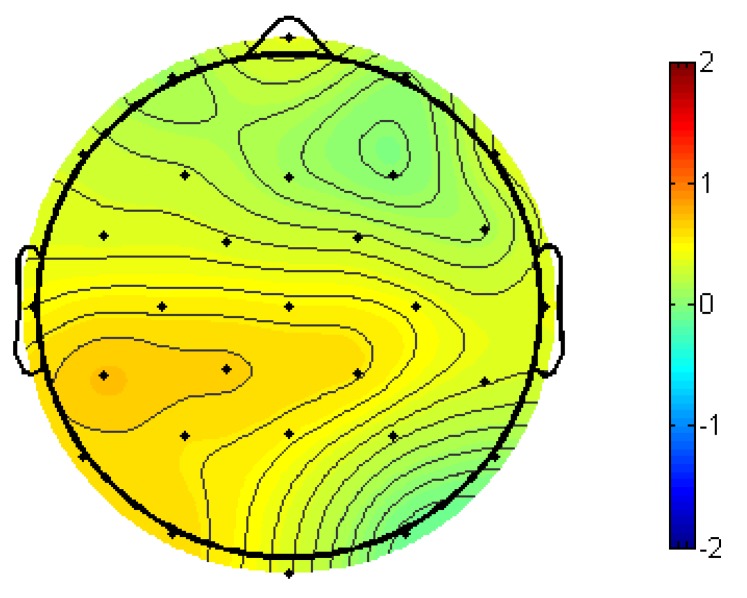
The 300–500-ms voltage map of L1 translation lexical relatedness priming effect for semantically unrelated targets (lexically unrelated translations–lexically related translations).

The main effect of interest was the whether L1 translation lexical relatedness influenced N400 processing of semantically unrelated L2 word pairs. To explore this effect, ERPs to semantically unrelated L2 targets were compared based on whether their L1 translations were lexically related or not. Across all participants, there were no significant differences between the N400 amplitudes of these two conditions, although semantically unrelated targets with lexically unrelated translations elicited slightly smaller N400s than semantically unrelated targets with lexically related translations (opposite direction priming). This can be seen in Figure 9 with the two semantically unrelated conditions; L2 targets with L1 lexically unrelated translations are shown in black and those with L1 lexically related translations are shown in red. The voltage map of the difference from 300–500 ms is shown in Figure 10. 

**Figure 11:**
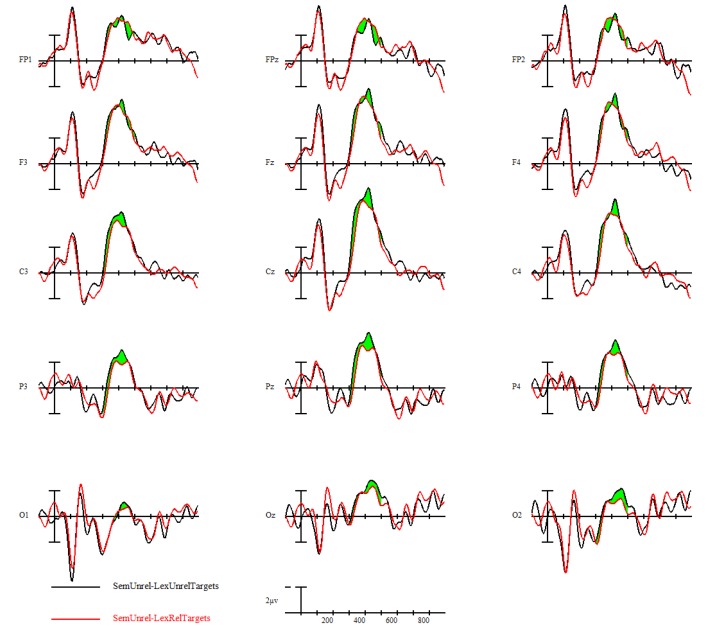
L1 translation lexical priming effect in low-attrition bilinguals (*N* = 12).

**Figure 12:**
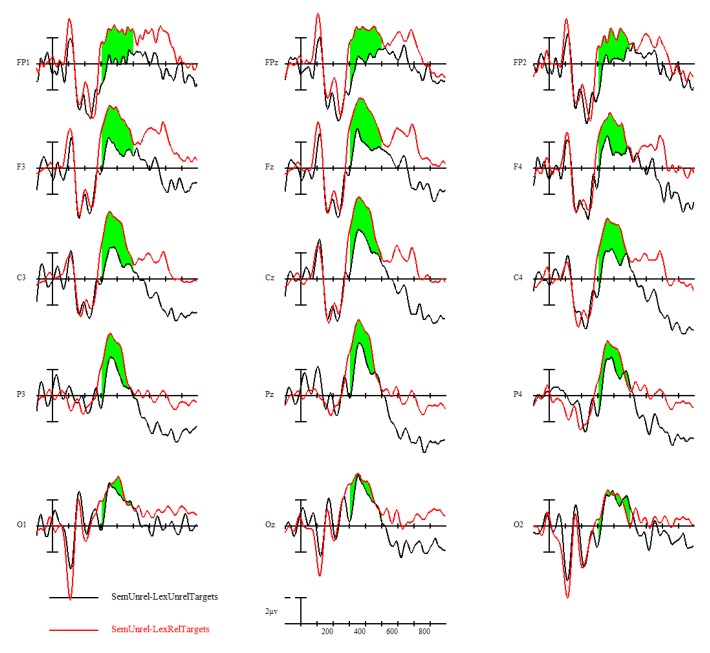
L1 translation lexical priming effect in high-attrition bilinguals (*N* = 8).

Despite this lack of main effect in the overall group, we were interested in seeing whether the low versus high L1 attrition groups showed differing priming effects of L1 translation lexical relatedness. In fact, the group comparisons indicated opposite priming directions; low L1 attrition bilinguals showed a priming effect in the typical direction (related targets eliciting smaller N400s than unrelated targets) (see Figure 11), but high L1 attrition bilinguals showed a reverse priming effect (unrelated targets elicited smaller N400s than related targets) (see Figure 12). This priming effect was not significant in the low L1 attrition group (*F*(1,11) = 0.4, *p* = 0.538), but was significant in the high L1 attrition group, *F*(1,7) = 11.37, *p* = 0.012. The opposing-direction priming effects across the two groups contributed to the overall lack of main effect in the entire bilingual sample. 

**Figure 13:**
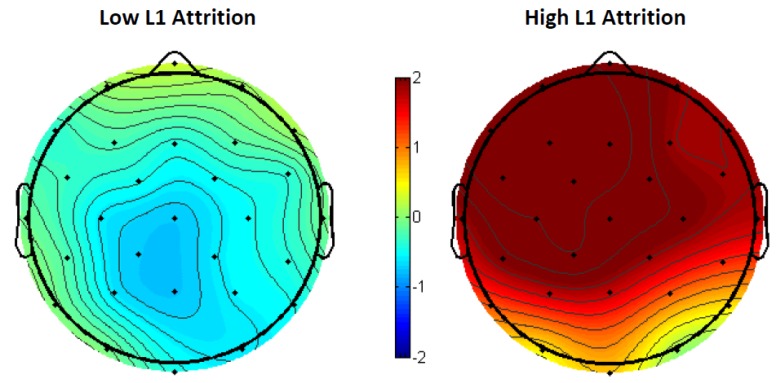
The 300–500-ms voltage maps of the L1 translation lexical relatedness priming effect (lexically unrelated translations–lexically related translations) in low/high L1 attrition bilinguals.

There was a significant interaction between the opposing L1 translation lexical priming effects of the low and high L1 attrition groups across all 15 electrodes (*F*(1, 299) = 61.78, *p* < 0.001), as shown in the voltage map (Figure 13) 

#### 3.3.4. N400 L1 Translation Lexical Priming Effect—Correlations with L1 Attrition and Its Factors

**Figure 14:**
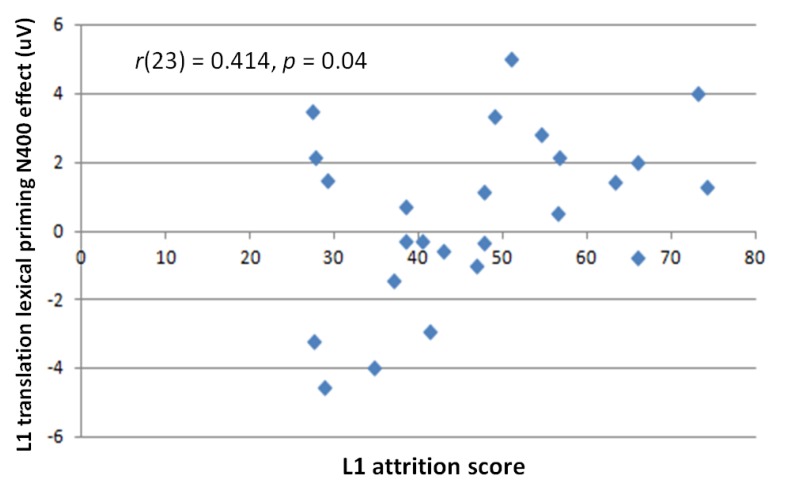
L1 translation lexical priming N400 effect against participant L1 attrition scores.

The low versus high L1 attrition group differences in critical comparison just mentioned suggested that L1 attrition was a factor in the presence of L1 translation effects. Correlation analysis between individual attrition scores and the N400 L1 translation lexical relatedness effect in semantically unrelated conditions was significant (*r*(23) = 0.414, *p* = 0.04) at electrode Pz (Figure 14), a centro-parietal electrode site where N400 priming effects are typically seen [45].

## 4. Discussion

In the present study, Spanish–English bilinguals with a range of L1 attrition levels and comparable high/fluent L2 proficiency were tested in an L2 only semantic judgment task. Unbeknownst to participants, some of the semantically unrelated L2 word pairs were lexically related in their L1 translations. We tested for effects of L1 attrition on potential influences of L1 translation lexical priming for these seemingly unrelated L2 word pairs to evaluate recent proposals of selectivity versus nonselectivity in bilingual lexical access. 

The pre-experiment screening guaranteed that all participants had high proficiency in L2 and comparable levels of L1 (similar L1 learning experience/environment) prior to L1 attrition. A composite L1 attrition score, derived from factors shown to contribute to language attrition, correlated significantly with participants’ L2–L1 translation ability as seen in the post-ERP translation task; low L1 attrition bilinguals outperformed high L1 attrition bilinguals on English-to-Spanish translation, indicating that our calculated L1 attrition score could be reliably used as a measure of L1 lexical access. The behavioral results did not reveal significant effects of L1 attrition for either individual correlation or by group. However, significant interactions between low and high L1 attrition groups from the ERP data suggest contributions of L1 attrition on L2 lexical access that align with the nonselective bilingual lexical access view. 

### 4.1. Insights from Behavioral Performance

The Spanish–English bilinguals completed the English semantic judgment task with high accuracy (85.7%), indicating a proficient level of L2 required for the study. L1 translation lexical relatedness affected RT and accuracy results when the L2 prime–target pairs were semantically unrelated (“sling” and “mushroom” or “cloud” and “floor”); participants performed worse (higher RT and lower accuracy) for these trials when the L1 translations were lexically related (“sling”/*honda* and “mushroom”/*hongo*) than unrelated (“cloud”/*nube* and “floor”/*piso*). Supporters of the bilingual nonselective lexical access view would argue that this reflects the activation of the non-target language during target language processing [11,12,13,14]. In other words, L1 activation interferes with L2 processing. Even though L2 prime and target pairs were semantically and lexically unrelated (requiring a “no” response), the lexical relatedness of their L1 translations impaired the bilingual’s ability to correctly reject the unrelated pair. 

However, since L1 attrition level did not significantly affect the behavioral results, Costa et al.’s selective model [3] can also explain this L1 translation lexical relatedness effect [3]. The poorer reaction time and accuracy performance for the unrelated L2 word pairs with lexically related L1 translations can be explained by the organization of the L2 lexicon. Connections between unrelated L2 words like “sling” and “mushroom” are established during L2 acquisition due to lexical similarities in their L1 translations (*honda* and *hongo*). Bilinguals selectively activate their L2 during the task, but have a harder time correctly rejecting certain semantically and lexically unrelated L2 pairs because of their learned associations (and not because of non-target language activation). 

### 4.2. Insights from ERP Results

Brain waves collected during the semantic judgment task revealed an N400 semantic priming effect in all bilinguals. Targets that were semantically related to their primes (e.g., “husband” primed by “wife” elicited attenuated N400s relative to targets that were not semantically related to their primes (e.g., “mushroom” primed by “sling”). The N400 semantic priming effect is an established ERP effect that reflects the ease of processing a word’s meaning when the previously seen word shares semantic overlap [45]. Combined with the high accuracy performance on the semantic judgment task and, most importantly, the lack of L1 attrition group differences in behavioral and ERP effect size, this N400 semantic priming effect indicates that the Spanish–English bilinguals in this study had comparable high proficiency/fluency in L2. 

We did not find an N400 effect of L1 translation lexical priming in the overall grand mean. However, this was explained by the opposite priming effect directions in the low versus high L1 attrition groups. For semantically unrelated target conditions, low L1 attrition bilinguals elicited a standard N400 translation lexical priming effect; targets whose L1 translations were lexically related with those of the prime elicited smaller N400 amplitudes than those whose translations were lexically unrelated [52]. The reverse was seen in the high L1 attrition group, and the interaction between the two groups was significant. Correlation analysis found a significant positive relationship between level of L1 attrition and L1 translation lexical priming effect; as L1 attrition increased, the priming effect decreased until it reversed. 

The significant interaction between low and high L1 attrition groups and their L1 translation lexical priming N400 effect, in conjunction with the significant positive correlation between individual L1 attrition scores and the effect size, provides evidence of L1’s influence on L2 processing. Greater L1 lexical access, attributable to lower L1 attrition levels, facilitates processing of L2 words if their L1 translations are lexically related to that of a previously seen word. This is in spite of the lack of overt relatedness between the prime and target. As the amount of L1 attrition increases, L1 translation lexical relatedness appears to inhibit target processing, resulting in larger N400s to translation related L2 words and reversal of a typical lexical priming effect. 

### 4.3. Explaining L1 Attrition and L1 Translation Lexical Priming Effect Reversal

Highly L1 attrited bilinguals elicited larger N400s to L2 target words whose L1 translations were lexically related to that of the primes. This may reflect the more effortful processing of L1 lexical items due to diminished access to the L1 lexicon as a result of L1 attrition, as smaller N400 amplitudes reflect ease of lexicosemantic processing [52]. However, how does that explain the larger N400 to targets than primes?

As considered within the framework of inhibitory models of bilingual processing, related lexical candidates (e.g., “cat”, “cab”, and “cap”) compete for activation during reading [53]. Such competition is settled due to the strongest activated lexical item inhibiting the other lexical candidates. Bilinguals with high levels of L1 attrition not only require more effort to activate their L1, but, once activated, are less effective at inhibiting competition between L1 words due to their disuse of L1. This leads to the compounding of activation between lexically related L1 translations for the seen L2 words as shown in the larger N400 to targets than to primes. This difficulty with competition inhibition is not the case for the low L1 attrited Spanish–English bilinguals, who have greater control and access to their L1 lexicon. These bilinguals are more efficient with L1 activation and L1 lexical candidate competition inhibition, such that lexically related L1 words facilitate processing as seen in the typical lexical priming effect. 

### 4.4. Replicating Thierry and Wu’s (2007) L1 Translation Lexical Priming Effects

Although low L1 attrition bilinguals displayed a L1 translation lexical priming N400 effect in the standard direction, the effect was not significant on its own and, therefore, did not replicate the results of Thierry and Wu’s study [14]. A few major differences in study material and design can address this: (1) L1 versus L1 script differences, (2) lexical repetition versus relatedness, and (3) L1 semantic priming in Chinese.

To the first point, Chinese and English differ in orthography and phonology (logographic script versus alphabetic script, respectively) to a greater extent than Spanish and English (both alphabetic scripts). Logographic languages elicit different ERPs (e.g., N170 lateralization) from alphabetic languages [54,55] and involve a different mapping of lexical units onto meaning [56,57]. In Chinese, logographs (characters) are mapped directly onto meaning, while Spanish requires an additional layer of processing where alphabetic symbols (letters) are mapped onto morphemes and words before semantic mapping. This difference suggests that the mechanisms for word processing between Chinese and English are distinct from those between Spanish and English. In Thierry and Wu’s Chinese–English bilinguals, L1 translation activation for L2 words involved separate lexical processing mechanisms due to the script differences. This dissociation could result in the larger L1 translation lexical priming N400 effect found in their study, as L1 lexical processing is more separate from L2 lexical processing, thereby reducing between-language competition [58]. Our Spanish–English bilinguals experienced more lexical competition between L1 and L2 due to the same script; activation of Spanish lexical items must compete with activation of English lexical items to a greater degree than in Chinese versus English. This increase between language competition may account for the smaller L1 translation lexical priming N400 effect seen in this study. 

Another consideration is the nature of L1 translation lexical priming between our experiment and that of Thierry and Wu [14]. Different Chinese words can share the same character such that the L1 translations are lexical repetitions. The present experiment utilized L1 lexical relatedness (shared initial orthography and phonology) since complete overlap in Spanish would indicate identical words, thereby rendering the semantic unrelated condition impossible. Lexical repetition (complete overlap) elicits a far greater priming effect than lexical relatedness (partial overlap), which could explain our results. 

The final difference is the presence of semantic priming in Thierry and Wu’s L1 translations that was absent in our L1 translations. The Chinese characters used in the previous study not only shared complete form and phonological overlap, but semantic overlap as well. For example, the Chinese character *huo* shared between both “ham” (*huo tui*) and “train” (*huo che*) has a meaning on its own: fire. When the Chinese–English bilinguals activated *huo che* to the target “train” after activation of *huo tui* to the prime “ham”, processing was facilitated not only by lexical repetition, but by meaning repetition as well. This additive semantic priming would further attenuate the N400, leading to a stronger priming effect [45]. 

### 4.5. Reducing Between-Language Competition: Evidence from Bimodal Bilinguals

As previously mentioned, our attenuated L1 translation lexical priming N400 effect relative to that of Thierry and Wu’s study could be due to the script similarity between our bilinguals’ L1 and L2. Spanish and English, by virtue of sharing the same script, compete for lexical activation during word processing. Such competition was seen in the increased reaction time to name L2 interlingual homographs (e.g., “pie” is “foot” in Spanish and a baked pastry in English) compared to L2 control words [59]. Shared morphology or phonology across alphabetic scripts can, therefore, interfere with target language activation. Despite not sharing the same script, between-language competition still exists between Chinese and English as seen in cross-language interference during language production tasks [11]. 

To better dissociate between L1 and L2 and reduce the between-language competition that may be attributed to reduced priming effects, we can turn to studies of bimodal bilinguals who use spoken/written and signed languages. Spoken and signed languages utilize separate perceptual systems during language comprehension (auditory for spoken languages and visual for signed languages), which results in diminished between-language competition relative to that between two spoken languages [60]. American Sign Language (ASL)–English bilinguals were shown to demonstrate similar N400 priming effects as Thierry and Wu’s results when visually presented with English words with implicitly phonologically (hand configuration, location, or movement) related or unrelated signed translations [61]. The distinct pathways to comprehension between the spoken English and visual signs leads to less lexical overlap (and less lexical competition), while still providing evidence for bilingual lexical nonselectivity. In bimodal bilinguals, parallel activation is posited to be a function of lateral lexical connections or top-down semantic to lexical connections between visual words and their signed translations [62].

### 4.6. Costa et al. versus Wu and Thierry 

The main goal of the present study was to evaluate the validity of a selective model of bilingual lexical processing [14] against the traditional nonselective view. L1 attrition was selected as a variable that could dissociate between the two due to the constraints of Costa et al.’s model [3] that L2 reorganization is established during L2 learning when L1 was the primary language of use. Therefore, degradation of the L1 due to attrition and the consequent reduced L1 lexical access should not influence L2 organization or processing. The Spanish–English bilinguals in this study all acquired L2 after learning L1, allowing for L1 lexical organization to influence L2 lexical organization if Costa and colleagues are correct [3]. However, all participants experienced some form of L1 attrition later on as they continued studying in an L2 environment. By this point, L2 lexical organization and, in particular, the grade-4–6-level stimuli used in this experiment, should no longer be influenced by L1. Per the model, L1 attrition should, therefore, not affect L2 processing. 

However, the ERP results do in fact show such an influence; when performing in an exclusively L2 language task, low L1 attrited bilinguals significantly differed from high L1 attrited bilinguals in their L1 translation lexical priming N400 effect. This effect size was significantly correlated with L1 attrition scores, with lower L1 attrition facilitating L2 target processing. This finding is compatible with a nonselective view of bilingual lexical access, where the nontarget language impacts target language processing. Differential L1 lexical access due to differential L1 attrition levels led to markedly different neural signatures of L2 processing. Our results, therefore, support Thierry and Wu’s original conclusion over Costa et al.’s reinterpretation. 

## 5. Conclusions

The present study tested for behavioral and ERP effects of L1 attrition on L2 processing to investigate the nature of bilingual lexical access. Manipulation of L1 attrition in an L2 semantic judgment task with implicit L1 translation relatedness allowed for the disentangling of two opposing views: Costa et al.’s model of selective L2 processing [3] and the nonselective, parallel L1/L2 activation view [11,12,13,14]. Our results revealed that Spanish–English bilinguals differed in their L2 processing as a function of their L1 attrition level. This was predicted only if bilingual lexical processing involved parallel activation of both nontarget and target languages and not by Costa et al.’s selective processing model. Thus, the findings corroborate the nonselective bilingual lexical access view. 

Additionally, while the behavioral results did not show L1 attrition effects, the ERP data did. This study, therefore, adds another line of support for the use of more sensitive cognitive neuroscience measures in linguistic research, which can uncover attributes of cognitive processing not revealed by behavior [33]. Influences of independent variables such as L1 attrition may manifest along the time course of word processing as seen in the present study, despite not eliciting behavioral effects. Altogether, ERP experiments in bilingual research offer a useful tool for elucidating competing views in the field.

## Figures and Tables

**Table 1:** The 2 × 2 stimuli conditions.

Conditions in 2 × 2 Design	Semantically Related	Semantically Unrelated
Translations Related	Wife–husband *Esposa–esposo*	Sling–mushroom *Honda–hongo*
Translations Unrelated	Moon–sun *Luna–sol*	Book–love *Libro–amor*

## Supplementary Materials

The following are available online at https://www.mdpi.com/2076-3425/9/6/126/s1, Table S1: Testing for nonselective bilingual lexical access using L1 attrited bilinguals.
