# Supplementary material for: Testing for Nonselective Bilingual Lexical Access Using L1 Attrited Bilinguals
Source: Brain Sci. 2019 Jun 1;9(6):126. doi: 10.3390/brainsci9060126 (PMC6628369; doi:10.3390/brainsci9060126)
Supplement: Supplementary file 1 [file brainsci-09-00126-s001.pdf]

Supplementary Materials: Testing for nonselective bilingual lexical access using L1 attrited bilinguals

English (L2) Prime-Target Pairs (with L1/Spanish Translations) in the Four Conditions

L2 Prime	Prime L1 Translation	L2 Target	Target L1 Translation	Condition
sting	*aguijon*	needle	*aguja*	Semantically Related/L1 Translation Lexically Related
garlic	*ajo*	sesame	*ajonjolí*	
shrub	*arbusto*	tree	*árbol*	
weapon	*arma*	armor	*armaduras*	
pocket	*bolsillo*	handbag	*bolso*	
horse	*caballo*	goat	*cabra*	
street	*calle*	alley	*callejón*	
load	*carga*	cargo	*cargamento*	
cart	*carreta*	stroller	*carriola*	
vest	*chaleco*	jacket	*chaqueta*	
waist	*cintura*	belt	*cinturon*	
kitchen	*cocina*	cook	*cocinera*	
spoon	*cuchara*	knife	*cuchillo*	
neck	*cuello*	body	*cuerpo*	
devil	*diablo*	god	*dios*	
shove	*empellón*	push	*empujon*	
nurse	*enfermera*	patient	*enfermo*	
wrapping	*envoltura*	shipping	*envio*	
writer	*escritor*	desk	*escritorio*	
wait	*espera*	hope	*esperanza*	
flower	*flor*	vase	*florero*	
cat	*gato*	kitten	*gatito*	
toy	*juguete*	game	*juego*	
spear	*lanza*	thrower	*lanzador*	
rain	*lluvia*	drizzle	*llovisna*	
tangerine	*mandarina*	apple	*manzana*	
sea	*mar*	sailor	*marinero*	
midnight	*medianoche*	noon	*mediodía*	
girl	*niña*	children	*niños*	
bread	*pan*	bakery	*panadería*	
blink	*parpadeo*	eyelid	*parpado*	
pastry	*pasteles*	cake	*pastel*	
scooter	*patin*	skate	*patines*	
duck	*pato*	turkey	*pavo*	
fight	*pelea*	danger	*peligro*	
toupee	*peluquín*	wig	*peluca*	
pickle	*pepinillo*	cucumber	*pepino*	
leg	*pierna*	feet	*pies*	
queen	*reina*	king	*rey*	
giggle	*risilla*	laughter	*risa*	
roar	*rugido*	noise	*ruido*	
salt	*sal*	sauce	*salsa*	
dryness	*sequedad*	drought	*sequia*	
shadow	*sombra*	hat	*sombrero*	
TV	*televisión*	phone	*teléfono*	
aunt	*tía*	uncle	*tío*	
work	*trabajo*	suit	*traje*	
fan	*ventilador*	window	*ventana*	
vineyard	*viñedo*	wine	*vino*	
skunk	*zorrillo*	fox	*zorro*	

L2 Prime	Prime L1 Translation	L2 Target	Target L1 Translation	Condition
water	*agua*	avocado	*aguacate*	Semantically Unrelated/L1 Translation Lexically Related
flag	*bandera*	bench	*banca*	
beard	*barba*	ship	*barco*	
homeless	*vagabundo*	wagon	*vagón*	
cheek	*cachete*	cub	*cacharro*	
pumpkin	*calabaza*	dungeon	*calabozo*	
face	*cara*	snail	*caracol*	
meat	*carne*	letter	*carta*	
ash	*ceniza*	dinner	*cena*	
tail	*cola*	hill	*colina*	
rabbit	*conejo*	advice	*consejo*	
heart	*corazón*	crown	*corona*	
room	*cuarto*	square	*cuadro*	
horn	*cuerno*	leather	*cuero*	
crow	*cuervo*	cave	*cueva*	
stairs	*escaleras*	shiver	*escalofrío*	
school	*escuela*	sculptor	*escultor*	
sword	*espada*	back	*espalda*	
mirror	*espejo*	wife	*esposa*	
stove	*estufa*	student	*estudiante*	
cricket	*grillo*	scream	*grito*	
shoulder	*hombro*	man	*hombre*	
bone	*hueso*	egg	*huevo*	
side	*lado*	brick	*ladrillo*	
madman	*loco*	mud	*lodo*	
wood	*madera*	mother	*madre*	
fear	*miedo*	honey	*miel*	
coin	*moneda*	monkey	*mono*	
orange	*naranja*	nose	*nariz*	
shotgun	*escopeta*	broom	*escoba*	
ear	*oreja*	gold	*oro*	
shovel	*pala*	word	*palabra*	
stick	*palo*	dove	*paloma*	
pants	*pantalón*	swamp	*pantano*	
umbrella	*paraguas*	couple	*pareja*	
chest	*pecho*	weight	*peso*	
stone	*piedra*	skin	*piel*	
silver	*plata*	dish	*plato*	
town	*pueblo*	bridge	*puente*	
door	*puerta*	port	*puerto*	
branch	*rama*	frog	*rana*	
week	*semana*	seed	*semilla*	
whistle	*silbido*	chair	*silla*	
smile	*sonrisa*	sound	*sonido*	
sip	*sorbo*	deafness	*sordera*	
tub	*tina*	ink	*tinta*	
bull	*toro*	tower	*torre*	
candle	*vela*	sailboat	*velero*	
deer	*venado*	poison	*veneno*	
trip	*viaje*	life	*vida*	

L2 Prime	Prime L1 Translation	L2 Target	Target L1 Translation	Condition
death	*muerte*	war	*guerra*	Semantically Related/L1 Translation Lexically Unrelated
clock	*reloj*	time	*tiempo*	
star	*estrella*	moon	*luna*	
bracelet	*pulsera*	necklace	*collar*	
bathroom	*baño*	soap	*jabón*	
trash	*basura*	mess	*revoltijo*	
drink	*bebida*	cup	*taza*	
ice	*hielo*	snow	*nieve*	
fabric	*tejido*	clothing	*vestimenta*	
nest	*nido*	cage	*jaula*	
loss	*extravío*	theft	*robo*	
warrior	*guerrera*	shield	*escudo*	
arrow	*flecha*	hunter	*cazador*	
thirst	*sed*	hunger	*hambre*	
elbow	*codo*	knee	*rodilla*	
bandage	*vendas*	ointment	*pomada*	
bubble	*burbuja*	ball	*pelota*	
grease	*sebo*	oil	*aceite*	
shell	*concha*	beach	*playa*	
drawing	*dibujo*	paint	*pintura*	
witch	*bruja*	ghost	*fantasma*	
lips	*labios*	kiss	*beso*	
doorbell	*timbre*	key	*llave*	
flight	*vuelo*	airplane	*avión*	
blouse	*blusa*	coat	*abrigo*	
socks	*calcetines*	shoe	*zapato*	
dessert	*postre*	sugar	*azúcar*	
hallway	*corredor*	ceiling	*techo*	
book	*libro*	library	*biblioteca*	
pepper	*pimienta*	carrot	*zanahoria*	
gum	*chicle*	candy	*dulce*	
weaver	*tejedor*	basket	*canasta*	
fire	*fuego*	heat	*calor*	
lunch	*almuerzo*	food	*comida*	
summer	*verano*	winter	*invierno*	
drunk	*borracho*	beer	*cerveza*	
bed	*cama*	pillow	*almohada*	
whale	*ballena*	fish	*pez*	
ant	*hormiga*	bee	*abeja*	
blanket	*cobija*	mattress	*colchón*	
tongue	*lengua*	throat	*garganta*	
tear	*lágrima*	eye	*ojo*	
finger	*dedo*	paw	*pata*	
gift	*regalo*	package	*paquete*	
tax	*impuestos*	wealth	*riqueza*	
wheel	*rueda*	car	*coches*	
smoke	*humo*	dust	*polvo*	
nephew	*sobrino*	sister	*hermana*	
rice	*arroz*	corn	*maíz*	
spring	*primavera*	season	*temporada*	

L2 Prime	Prime L1 Translation	L2 Target	Target L1 Translation	Condition
tooth	*diente*	building	*edificio*	Semantically Unrelated/L1 Translation Lexically Unrelated
driver	*chofer*	doll	*muñeca*	
costume	*disfraz*	claw	*garras*	
axe	*hacha*	forest	*bosque*	
wind	*viento*	bucket	*cubeta*	
dress	*vestido*	hug	*abrazo*	
pencil	*lápiz*	drum	*tambor*	
tie	*vínculo*	napkin	*servilleta*	
bath	*bañada*	milk	*leche*	
city	*ciudad*	crying	*llanto*	
shore	*orilla*	rope	*mecate*	
trunk	*trompa*	saving	*ahorro*	
root	*raíz*	silk	*seda*	
sky	*cielo*	entrails	*entrañas*	
earth	*tierra*	hair	*cabello*	
arm	*brazo*	thief	*ladrón*	
chain	*cadena*	clothes	*ropa*	
lounge	*salón*	pain	*dolor*	
deck	*cubierta*	chalk	*gis*	
cloud	*nube*	floor	*piso*	
fuel	*combustible*	neighbor	*vecina*	
drawer	*cajón*	boat	*embarcación*	
thunder	*trueno*	truth	*verdad*	
glasses	*anteojos*	sweat	*sudor*	
bird	*pájaro*	son	*hijo*	
rug	*tapete*	grass	*pasto*	
scratch	*rasguño*	teacher	*maestro*	
top	*cumbre*	country	*país*	
money	*dinero*	step	*paso*	
lie	*mentira*	sign	*señal*	
carpet	*alfombra*	feather	*pluma*	
table	*mesa*	boots	*botas*	
skull	*calavera*	dog	*perro*	
molar	*muela*	trap	*trampa*	
belly	*panza*	leaf	*hoja*	
bedroom	*recámara*	butter	*manteca*	
utensil	*cubierto*	mustache	*bigote*	
ankle	*tobillo*	lights	*luces*	
mouse	*ratón*	employee	*empleado*	
store	*tienda*	potato	*papa*	
plum	*ciruela*	shirt	*camisa*	
twin	*gemelo*	wall	*muro*	
name	*nombre*	apron	*delantal*	
marriage	*matrimonio*	nap	*siesta*	
blood	*sangre*	goods	*bienes*	
cleaning	*limpia*	pen	*bolígrafo*	
love	*amor*	recipe	*receta*	
yawn	*bostezo*	flame	*llama*	
fang	*colmillo*	puppet	*títere*	
grape	*uva*	head	*cabeza*	
